# Withholding and withdrawal of care in the ICU of Eastern France modalities and families feeling

**DOI:** 10.1017/S1478951526101850

**Published:** 2026-02-19

**Authors:** Cindy Chauchard, Antoine Goury, Bruno Lafon, Laurent Poiron, Salvatore Muccio, Magdalena Szczot, Georges Simon, Quentin Georges, Vincent Castelain, Alice Duvivier, Lukshe Kanagaratnam, Vincent Legros

**Affiliations:** 1 Anesthesiology, Critical Care and Perioperative Medicine, University Hospital of Reims, Reims, France; 2 Medical Intensive Care Unit, University Hospital of Reims, Reims, France; 3 Intensive Care Unit, Polyclinique Reims-Bezannes, Bezannes, France; 4 Intensive Care Unit, Hospital of Châlons-en-Champagne, Châlons-en-Champagne, France; 5 Department of Anesthesiology and Critical Care, University Hospital of Strasbourg, Strasbourg, France; 6 Intensive Care Unit, Hospital of Troyes, Troyes, France; 7 Intensive Care Unit, Hospital of Haguenau, Haguenau, France; 8 Service de médecine intensive – réanimation, Hôpital de Hautepierre, Hôpitaux Universitaires de Strasbourg, Strasbourg, France; 9 Fédération de Médecine Translationnelle de Strasbourg, Université de Strasbourg, Strasbourg, France; 10 Department of Clinical Research, University Hospital of Reims, Reims, France; 11 Université de Reims Champagne-Ardennes, VieFra, Reims, France

**Keywords:** ICU, family relationship, withdrawal of life-sustaining therapies, observational study, Multicenter study

## Abstract

**Background:**

Withholding or withdrawing life-sustaining therapies (WLST) was introduced in France in 2005 through the Leonetti law to prevent futile treatments and “unreasonable obstinacy.” In France, WLST decisions affect 8.5–14% of ICU patients, according to the literature. The 2016 Claeys–Leonetti law updated the previous legislation, but debates surrounding end-of-life care persist.

**Methods:**

To describe WLST patients and practices under current legislation, we conducted a multicenter, prospective, observational study in ICUs across Eastern France. Eligible adult patients facing WLST decisions were included, requiring written consent from the patient or a trusted person. Patients were followed for 1 month. We described the decision-making process and assessed family satisfaction using the FS-24R-ICU questionnaire.

**Results:**

Between May 3rd and October 3rd, 2023, 73 patients were included (mean age 69 years). The majority of admissions were medical (72.7%), and 50.7% of patients had neurological impairments. ICU staff initiated WLST discussions primarily due to poor survival or quality of life prospects. Only 12.5% of patients had written advance directives, and 59.1% had designated a trusted person. External consultation was not involved in 19.1% of decisions. Families were informed in 91.7% of cases. Decisions to withhold therapies occurred in 68.1% of cases, with resuscitation during cardiac arrest being the most commonly withheld intervention (98.0%). Treatment withdrawal occurred in 31.9% of cases. Family satisfaction was generally positive.

**Conclusions:**

WLST management in Eastern French ICUs is partially compliant with the Claeys–Leonetti law. Improved law application and public awareness could enhance end-of-life care management in France.

## Background

Critical care has advanced significantly in recent years, allowing for the treatment of increasingly severe cases through improved medical practices. Admissions to Intensive Care Units (ICUs) have also expanded to include older patients with a higher burden of comorbidities (Sprung et al. 2019). Nevertheless, ethical concerns persist regarding the appropriateness of care delivered to these patients. While such care is often technically feasible, it may sometimes be disproportionate, unreasonable, futile, or unwanted by the patient (even if only expressed by the family).

In response, both European and French legislative frameworks have evolved, given the ongoing debate around end-of-life care. In France, the withholding or withdrawal of life-sustaining therapies (WLST) was formally established by the Leonetti law in 2005 (Loi n°2005-370, 2005), later amended by the Claeys–Leonetti law in 2016 (Loi n°2016-87, 2016). The 2016 amendment reinforced patient autonomy by prioritizing their wishes. When patients cannot express preferences, clinicians must first consult written advance directives, which are legally binding unless deemed manifestly inappropriate or in emergency situations. If no directives exist, the opinion of a designated healthcare proxy, or otherwise the family, must be sought. The law also requires that any WLST decision be documented following a formal collegial deliberation process involving the medical team and, when feasible, an external independent consultant. Relatives must be informed of the decision and its rationale.

A key innovation introduced by the 2016 law is the right to continuous deep sedation until death, under strict conditions, for patients with refractory suffering or those opting for WLST.

Currently, WLST decisions account for approximately 9–14% of ICU admissions in France. Prior to legislative changes, the EPILAT group aimed to investigate the procedural aspects of WLST in French ICUs and their adequacy with the existing law (2015). It reported WLST implementation in 14% of ICU patients. Few studies have evaluated WLST practices following the 2016 Claeys–Leonetti law. In patients over 80 years, WLST decisions occurred in 27.8% of cases. Between 2016 and 2018, WLST decisions were recorded in 6% of trauma patients admitted to 14 French Level I or II trauma centers included in the Traumabase Registry (Haddam et al. 2022). LAT group data from 2018 analyzed WLST decision-making in emergency departments (Douplat et al. 2020). However, high-quality recent data evaluating adherence to the current law and management practices remain scarce.

This study aimed to assess WLST decision-making and management in ICUs following the 2016 legislative changes and to evaluate the psychological impact of these decisions on patients’ relatives.

## Methods

### Study design, aim, and setting

We conducted a prospective, multicenter, observational study in ICUs across Eastern France, starting on April 3, 2023 (ClinicalTrials.gov Identifier: NCT05465187; registered July 19, 2022). All types of ICUs (medical and surgical) were invited to participate.

The primary objective was to describe current practices and patient characteristics associated with WLST decisions. Secondary objectives included evaluating adherence to the French legal framework (Claeys–Leonetti law, February 2, 2016), particularly in terms of the presence of a declared trusted person, the presence of a collegial meeting that takes into account healthcare professionals, the opinion of an external consultant physician, and information provided to the patient or their relatives.

### Participants

All adult patients admitted to the ICU for whom a decision of withholding or withdrawing of life-sustaining therapy is taken during the study period were eligible for inclusion.

Exclusion criteria included patients for whom a WLST decision had been made prior to the study period, refusal by the patient or their family to participate in the study, patients under the age of 18, and patients under legal protective measures. Each patient was followed up for 1 month from the date of inclusion.

### Data collection

Collected data included demographic information (age, sex), medical history, admission type (medical, trauma, elective or emergency surgery), and severity scores (Sepsis-related Organ Failure Assessment [SOFA], Index de Gravité Simplifié II [IGS II]). We also recorded the presence of organ failure and outcomes at ICU discharge and at day 28 (see Supplementary Data 2).

WLST process data were collected using a standardized form issued by the French Society of Anesthesiology and Critical Care.

We also assessed family satisfaction using the revised FS-24R-ICU questionnaire (Family Satisfaction with ICU – 24 Revised Questionnaire) (Wall et al. 2007). This is a validated survey used to hear opinions about patient family member’s admission to the ICU. It is divided into 2 parts, the first one regarding satisfaction with care (patient consideration, relatives supporting, the ICU staff, the waiting room, the ICU care), the second one interesting satisfaction with decision taking around care (information needs). For each item, response options include a color “faces” scale to visually represent different levels of satisfaction (from very dissatisfied to completely satisfied).

Each participating center appointed an intensivist as the study investigator, responsible for patient inclusion and data entry. eCRFs were used, and all data were centralized at the University Hospital of Reims.

### Statistical analysis

Quantitative variables are expressed as mean ± standard deviation or as median with interquartile range, depending on the distribution. Qualitative variables are expressed as frequencies and percentages. Univariate analysis was performed using the Student’s *t*-test, Mann–Whitney *U* test, Chi-square test, or Fisher’s exact test, as appropriate.

Results from the FS-24R-ICU questionnaire are presented as histograms, with each item scored on a 1–5 scale and transformed into a 0–100% scale. The distribution of responses is illustrated using color-coded bars.

All analyses were conducted using SAS version 9.4 (SAS Institute Inc., Cary, NC, USA).

## Results

**Figure 1.**
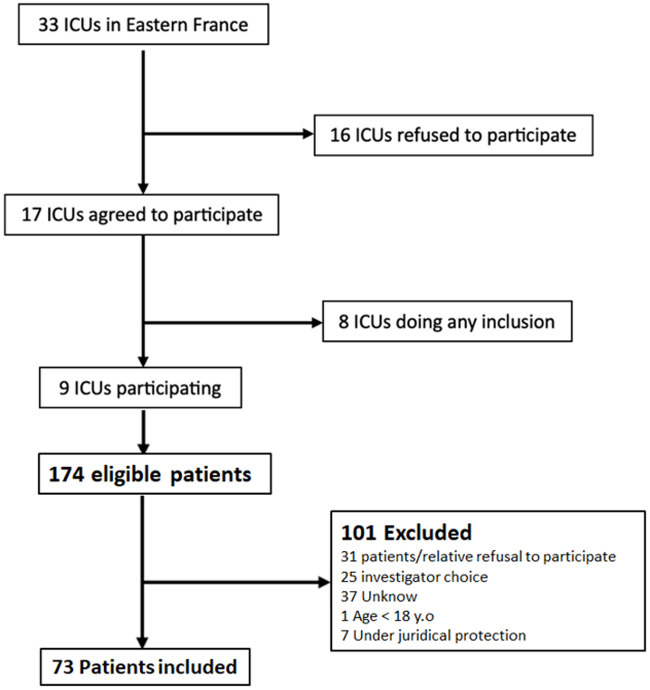
Flow chart.

Of the 33 ICUs in Eastern France, 17 agreed to participate, and 9 ultimately included patients (Figure 1, Supplementary Data 5). Between May 3 and October 3, 2023, a total of 73 patients were enrolled.

### Baseline characteristics

The patients for whom a WLST decision was made had a mean age of 69 years (95% CI [62–76]), with a nearly equal gender distribution. A history of active cancer was present in 31.5% of patients, and chronic respiratory failure was the most common chronic organ dysfunction (11%, 8/73).

The majority of admissions were medical (72.7%, 53/73). Surgical admissions (emergency or elective) accounted for 17.8% (13/73), and trauma-related admissions for 9.6% (7/73).

The most common organ failures were neurological impairment (50.7%, 37/73), circulatory failure (45.2%, 33/73), and acute or chronic kidney injury (35.2%, 26/73). A brain injury was diagnosed at admission or during hospitalization in 41.1% of cases (Tables 1 and 2).

**Table 1.** Baseline characteristics

	*n* = 73
Age (years)	69 [62; 76]
Sex, male, *n* (%)	42 (57.5%)
**Medical history**	
Active cancer, *n* (%)	23 (31.5%)
Chronic respiratory failure, *n* (%)	8 (11.0%)
Chronic heart failure, *n* (%)	4 (5.5%)
Chronic renal failure, *n* (%)	3 (4.1%)
**Reason for admission**	
Medicine, *n* (%)	53 (72.7%)
Trauma, *n* (%)	7 (9.6%)
Planned surgery, *n* (%)	3 (4.1%)
Urgent surgery, *n* (%)	10 (13.7%)
Existence of a brain injury visible in imaging	
(at the admission or occurring during hospitalization), *n* (%)	30 (41.1%)
**Organ failure^a^**	
Neurological failure, *n* (%)	37 (50.7%)
Circulatory failure, *n* (%)	33 (45.2%)
Kidney failure, *n* (%)	26 (35.2%)
Respiratory failure, *n* (%)	19 (26.0%)
Hematologic failure, *n* (%)	11 (15.1%)
Liver failure, *n* (%)	7 (9.6%)
SOFA at ICU admission	9 [6; 11]
SOFA at the day of WLST decision	8 [4; 10]
SAPS II	61 [49; 70]

SOFA = Sepsis-related Organ Failure Assessment; WLST = withdrawal of life sustaining therapies; SAPS = Simplified Acute Physiology Score. Data expressed in median [interquartile] or number (percentage).

a Neurological Failure, Glasgow Coma Scale < 13; Circulatory failure MAP < 65 mmHg or norepinephrine; Kidney Failure, KDIGO ≥ 1; Respiratory Failure, ventilatory support (Mechanical ventilation, NIV, NFNC), Hematologic failure WBC < 1 G/L or Platelets < 20 000 mm^3^; Liver failure rPT < 50% and/or bilirubin > 50 µmol/L.

**Table 2.** Results about WLST decision-making process

	*n* = 73
**Reason for initiating discussion**	
Situation of therapeutic failure, *n* (%)	21 (28.8%)
Unfavorable issue in terms of prolonged life and/or quality of life, *n* (%)	36 (49.3%)
Favorable issue but increasing therapeutics may be irrational, *n* (%)	13 (17.8%)
Request from medical staff or relatives, *n* (%)	3 (4.1%)
Available advance directives, *n* (%)	9 (12.5%)
Designated trusted person, *n* (%)	42 (59.1%)
**Discussion undertaking**	
The patient himself, *n* (%)	2 (2.7%)
Patient’s relatives/trusted person, *n* (%)	6 (8.2%)
Intensive care staff, *n* (%)	65 (89.0%)
**Members participating to discussion**	
Physicians from the unit, *n* (%)	71 (98.6%)
Patient’s nurse, *n* (%)	54 (75.0%)
Patient’s nurse’s aid, *n* (%)	28 (38.9%)
Residents, *n* (%)	55 (76.4%)
Medical students, *n* (%)	20 (27.8%)
Health manager, *n* (%)	18 (25.0%)
Physiologist, *n* (%)	4 (5.6%)
General practitioner, *n* (%)	2 (2.8%)
Physicians or surgeons taking care of the patient, *n* (%)	20 (27.8%)
**External consultant, *n* (%)**	**59 (81.9%)**
Intensivist from in another unit but working in the same center, *n* (%)	7 (11.9%)
Medical physician linked to reason for admission, *n* (%)	28 (47.5%)
Medical physician without link to reason for admission, *n* (%)	12 (20.3%)
Surgeon linked to reason for admission, *n* (%)	12 (20.3%)
External consultant agrees to decision, *n* (%)	57 (96.7%)
**Patient’s relatives informed about the WLST decision, *n* (%)**	66 (91.7%)
If yes, they support the decision, *n* (%)	65 (98.5%)
If yes, they want to be present at the moment of the death, *n* (%)	31 (44.3%)
**WLST decision**	
Withholding one or several treatments, *n* (%)	49 (68.1%)
Withdrawing treatments, *n* (%)	23 (31.9%)
**Evolution of the patient**	
Patient died in ICU, *n* (%)	52 (71.2%)
Patient alive at day 28, *n* (%)	14 (19.2%)

Data expressed in median [interquartile] or number (percentage).

### WLST management

In 89.0% of cases (65/73), discussions regarding WLST were initiated by the ICU medical team. The primary reason cited was an expected poor prognosis in terms of survival and/or quality of life (49.3%, 36/73), while 28.8% (21/73) involved therapeutic failure.

Only 12.5% (9/73) of patients had written advance directives, although 59.1% (42/73) had designated a trusted person.

**Figure 2.**
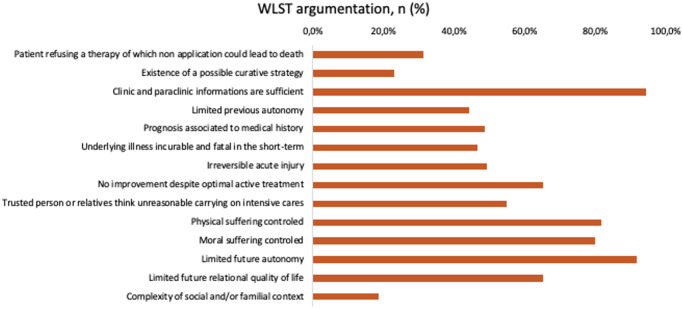
WLST argumentation.

The main criteria justifying the WLST decision, as reported by ICU teams, were the availability of adequate clinical and paraclinical information and the anticipation of poor future autonomy (Figure 2). Physical and psychological suffering were also appropriately managed in 81.1% and 80.0% of cases, respectively.

This figure shows the reasons cited by ICU teams to justify a WLST decision. The most frequently reported arguments included the availability of sufficient clinical and paraclinical data, the anticipated limitation of the patient’s future autonomy, and the effective management of physical and moral suffering. Medical justifications were predominant compared to social considerations or explicit wishes expressed by the patient or their relatives.

Decision-making discussions most frequently involved ICU physicians (98.6%), residents (76.4%), and nurses (75.0%). External consultants participated in 81.9% of cases (59/73), most often specialists relevant to the patient’s condition (47.5%), and agreed with the team in charge of the patient in 96.7% of cases.

The patients’ relatives were informed in 91.7% of cases (66/73) and in most cases agreed with the medical team (95.5%).

**Figure 3.**
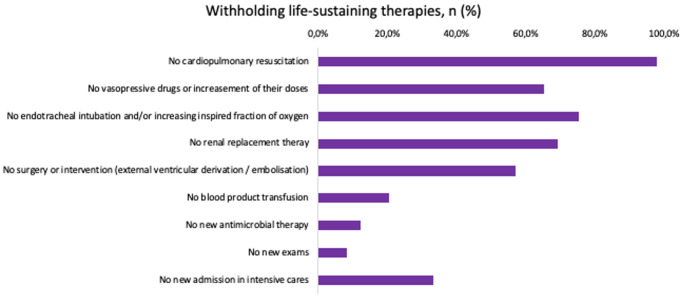
Withholding life-sustaining therapies.

Decisions to withhold life-sustaining treatments accounted for 68.1% (49/73), with the most commonly withheld intervention being resuscitation during cardiac arrest (98.0%) (Figure 3).

This figure illustrates the specific interventions that were withheld as part of decisions to limit life-sustaining treatment. The most commonly withheld therapies included cardiopulmonary resuscitation, vasopressor administration or dose escalation, mechanical ventilation or oxygenation support, and renal replacement therapy. More rarely, decisions involved the absence of surgery, transfusion, antimicrobial therapy, further diagnostic tests, or re-admission to the ICU.

**Figure 4.**
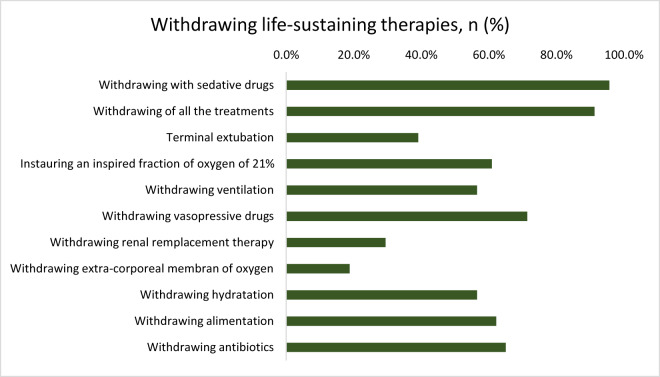
Withdrawing life-sustaining therapies.

Withdrawal decisions were made in 31.9% (23/73), and in 91.3% (21/23), all therapeutic interventions were discontinued. In nearly all of these cases (95.7%, 22/23), patients received deep and continuous sedation until death, in accordance with legal requirements (Figure 4).

This figure shows the different modalities of treatment withdrawal implemented as part of end-of-life decisions. The most common were the administration of sedative drugs, global treatment withdrawal, terminal extubation, and the cessation of vasopressors, hydration, alimentation, or oxygenation support. Less frequent interventions included the withdrawal of renal replacement therapy, extracorporeal oxygenation, and antibiotics.

### Family satisfaction

**Figure 5.**
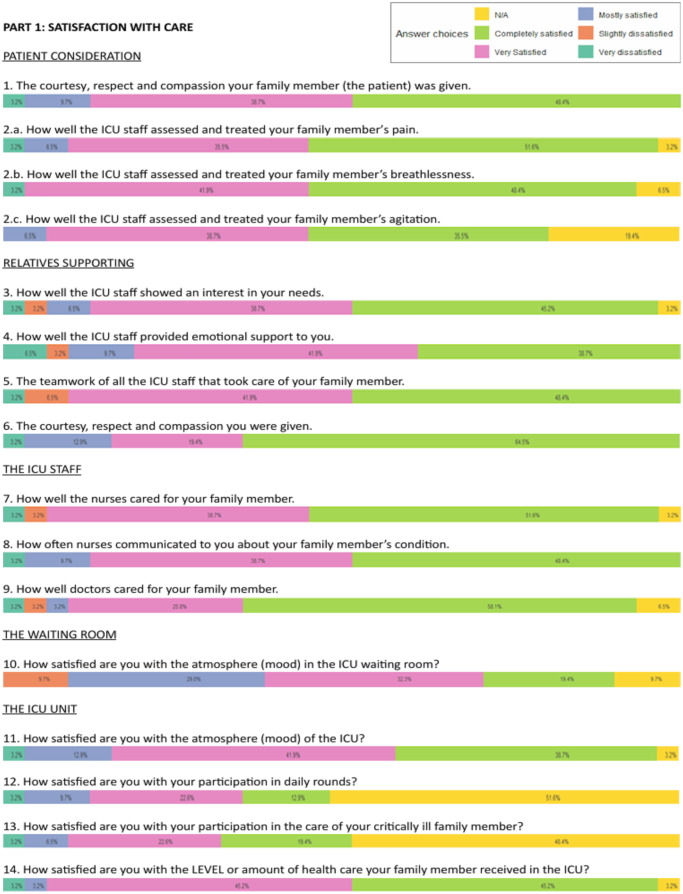
Satisfaction with care part 1.

**Figure 6.**
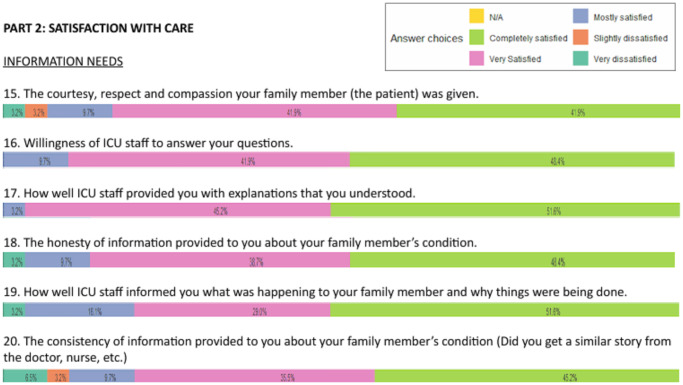
Satisfaction with care part 2.

Questionnaires were completed by the relatives of 32 out of 44 patients (72.7%). Overall satisfaction was high (Figures 5, 6, and supplemental figure). The lowest-scoring areas included:

- time available for families to express concerns and receive answers (rated “definitely inadequate” by 3.2%, and “slightly inadequate” by 9.7%),
- waiting room atmosphere (9.7% slightly dissatisfied), and
- frequency of communication with nurses (3.2% very dissatisfied, 9.7% slightly dissatisfied).

Most respondents were spouses or children of the patient, with diverse educational backgrounds. Notably, 75.0% had never previously experienced the ICU setting as a relative of a patient.

Figures highlighting the different aspects of family satisfaction.

## Discussion

### Main findings

Our multicenter study included various types of ICUs (medical, surgical, and specialized surgical) and institutions (university hospitals, non-university hospitals, and private clinics). It aimed to assess the implementation of WLST decisions in accordance with the Claeys–Leonetti law (2016). Our findings highlight notable heterogeneity in practices, with some deviations from legal requirements, raising concerns about healthcare providers’ understanding of the legislation.

For instance, in 20% of cases, external consultants were not involved – undermining the law’s requirement for collegial decision-making. Although the rate of patients with advance directives was higher than in previous studies, it remains low (12.5%), underscoring the need to enhance public awareness.

Conversely, the use of deep sedation until death in withdrawal cases was widespread and consistent with legal and ethical guidelines. We did not collect data on sedation details or terminal extubation, as these practices are already well-documented in the literature (Robert et al. 2017; Le Dorze et al. 2023).

A decade ago, Villers et al. (2010) emphasized the need for tools to help ICU physicians evaluate and improve their practices. However, to our knowledge, no French study to date has evaluated family satisfaction in the context of WLST decisions. In contrast, Hartog et al. (2015) assessed relatives’ perceptions and emotional burden in Germany in 2011, and similar studies exist in the United States. Our 2023 findings in France are reassuring.

We did not specifically study conflicts related to WLST decisions, though existing data on this topic are available (Giabicani et al., 2023).

### Comparison with literature

Numerous studies worldwide and in France have explored WLST practices (Azoulay 2008; Lobo et al. 2017; Avidan et al. 2021). In the 1997 French ICU study by Ferrand et al., WLST decisions were taken by the full medical team (54%) or without nursing staff (34%), and in 12% of cases by a single physician – practices no longer deemed acceptable under current law. This evolution reflects the growing adoption of collegial processes introduced by the Leonetti laws.

In 2013, the EPILAT group found that external consultants were involved in only 46% of WLST decisions. In contrast, our study reports 82%, suggesting improved compliance since the 2016 legislation. However, disparity persists: the LAT group found that only 39.3% of WLST decisions in emergency departments involved external consultants. This discrepancy may reflect stronger training and familiarity with WLST requirements among intensivists compared to other specialties.

Advance directives were available in 12.5% of cases in our cohort, compared to 1.3% in EPILAT’s 2013 findings. Still, public awareness remains insufficient. A 2022 CNSPFV survey reported that 56.8% of French people were unfamiliar with the term “advance directives,” and among those who were aware, 80.9% had not drafted them, and 29.3% did not know how. A standard form is available on the French National Authority for Health (HAS) website, and such directives are legally binding without delay since 2016 (HAS (Haute Autorité de Santé) 2016; Le Dorze et al. 2019). Several barriers to drafting advance directives likely persist – especially among critically ill patients (Andreu et al. 2018).

Family communication has also improved since 1997, when only 44% were informed about WLST decisions. In our study, nearly all relatives were informed. It is important to emphasize that, under French law, families serve in an advisory – not decision-making – role. Nonetheless, 13% of relatives reported insufficient time to ask questions and express concerns, which could change physician’s attitude if they really know relatives’ minding. Effective communication between clinicians and families remains essential for high-quality end-of-life care in ICUs (Curtis et al. 2001, 2002). Notably, a randomized trial by Robin et al. (2021) demonstrated that an informational brochure about families’ roles in end-of-life care significantly reduced Post-Traumatic Stress Disorder symptoms among bereaved relatives (RR = 1.8; 95% CI: 1.2–2.7).

These results suggest that communication could be further optimized to ensure that families feel heard, even within the legal framework.

### Limitations

Several limitations should be acknowledged. First, the number of included patients was lower than expected, despite an extended enrollment period. We should restate that of the 33 ICUs in Eastern France, 17 agreed to participate, but only 9 included patients. Moreover, emotional and logistical challenges often prevented patient inclusion – some families felt uncomfortable, or investigators hesitated to approach them or lacked time. Second, some data were missing or incomplete (12 FS-24R-ICU survey not completed for instance). The large number of patients not included, whether for unknown reasons or at the investigator’s discretion, is a real issue that creates a significant selection bias. Finally, generalizability is limited, as a large portion of patients were enrolled at the University Hospital of Reims.

## Conclusions

In Eastern French ICUs, WLST decisions are not always fully compliant with current legislation, particularly regarding the mandated involvement of external consultants. The low prevalence of advance directives continues to hinder ethical, patient-centered decision-making.

Although early family feedback is generally positive, further efforts are needed to improve public awareness, legal adherence, and communication practices – while respecting the anonymity of each other – ensuring decisions align with patients’ values and preferences.

## Supporting information

10.1017/S1478951526101850.sm001 Chauchard et al. supplementary material 1 Chauchard et al. supplementary material

10.1017/S1478951526101850.sm002 Chauchard et al. supplementary material 2 Chauchard et al. supplementary material

## Data Availability

The raw supporting the conclusion of this article will be made available by the authors upon reasonable request by sending an email to vlegros@chu-reims.fr.

## Supplementary material

The supplementary material for this article can be found at https://doi.org/10.1017/S1478951526101850.
