# Supplementary material for: Withholding and withdrawal of care in the ICU of Eastern France modalities and families feeling
Source: Palliat Support Care. 2026 Feb 19;24:e62. doi: 10.1017/S1478951526101850 (PMC13166340; doi:10.1017/S1478951526101850)
Supplement: Chauchard et al. supplementary material 1 — Chauchard et al. supplementary material [file S1478951526101850sup001.docx]

***Supplemental Figure .*** Decision making process


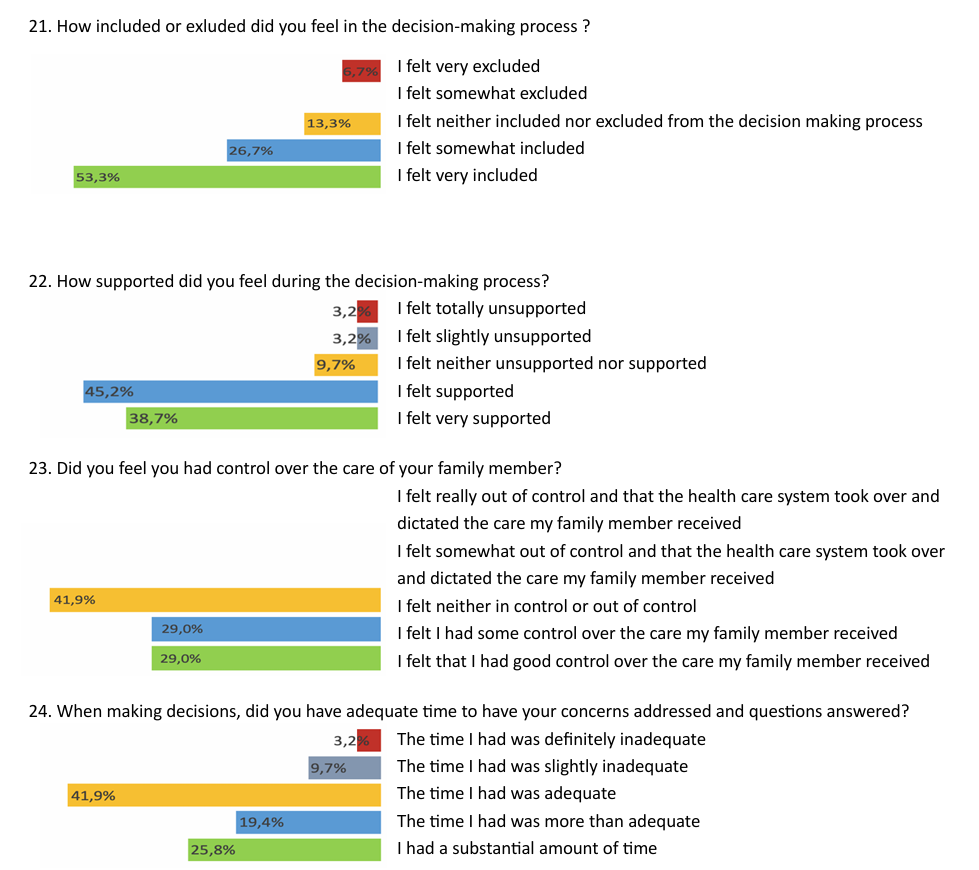


*Figure of the perception of the decision-making process*
