# Supplementary material for: Withholding and withdrawal of care in the ICU of Eastern France modalities and families feeling
Source: Palliat Support Care. 2026 Feb 19;24:e62. doi: 10.1017/S1478951526101850 (PMC13166340; doi:10.1017/S1478951526101850)
Supplement: Chauchard et al. supplementary material 2 — Chauchard et al. supplementary material [file S1478951526101850sup002.docx]

***Supplementary data 2.* eCRF**

**Date of ICU admission:** …../…../……….

**Date of WLST decision:** …../…../……….

**Age:** _____ **Sex:** ❒ Male ❒ Female

**Medical history: ❒** Cancer ❒ Metastatic cancer ❒ Malignant hemopathy

❒ HIV ❒ Cirrhosis (Child B/C) ❒ Chronic kidney failure ❒ Chronic kidney failure with RRT

❒ Chronic respiratory failure (*if yes:* with LTOT: ❒ yes / ❒ no) ❒ Chronic heart failure

**Reason for admission:** ❒ Medicine ❒ Trauma ❒ Poisoning ❒ Planned surgery ❒ Urgent surgery

**Existence of a brain injury** (at the admission or occurring during the hospitalization): ❒ Yes ❒ No

**Organ failure: ❒** Respiratory failure ❒ Neurological failure ❒ Circulatory failure ❒ Kidney failure ❒ Liver failure ❒ Hematologic failure

SOFA at the day of inclusion: _____ SOFA at the day of WLST decision: _____ SAPS II: _____

**Reason for initiating discussion:**

**❒** Situation of therapeutic failure

**❒** Unfavorable issue in terms of prolonged life and/or quality of life

**❒** Favorable issue but increasing therapeutics may be irrational

**❒** Request from medical/paramedical staff or patient’s relatives

**Available written advance directives:** ❒ Yes ❒ No

**Designated trusted person:** ❒ Yes ❒ No

**Discussion undertaking: ❒** The patient himself **❒** Patient’s relatives/trusted person

**❒** ICU medical staff **❒** ICU paramedical staff **❒** Medical staff from other specialties

**Members participating to discussion:**

**❒** Physicians from the unit **❒** Patient’s nurse **❒** Another nurse

❒ Health manager **❒** Patient’s nurse’s aid  **❒** Other nurse’s aid

**❒** Residents **❒** Medical students **❒** Paramedical students

**❒** Physiologist ❒ Psychologist ❒ General practitioner

❒ Physicians or surgeons taking care of the patient

**WLST argumentation:** *Agree / Disagree*

- - Patient refusing a therapy which non-application could lead to death

❒ ❒

❒ ❒

❒ ❒

❒ ❒

❒ ❒

❒ ❒

❒ ❒

❒ ❒

❒ ❒

❒ ❒

❒ ❒

❒ ❒

❒ ❒

❒ ❒

- - Existence of a possible curative strategy
  - Clinical and paraclinical information are sufficient
  - Limited previous autonomy
  - Prognosis associated to medical history
  - Underlying illness incurable and fatal in the short-term
  - Irreversible acute injury
  - No improvement despite optimal active treatment
  - Trusted person or relatives think unreasonable carrying on intensive cares
  - Physical suffering controlled
  - Moral suffering controlled
  - Limited future autonomy
  - Limited future relational quality of life
  - Complexity of social and/or familial context

**External consultant: ❒** Yes **❒** No

*If yes:*  ❒ Intensivist from another unit but working in the same hospital

❒ Intensivist from another hospital

❒ Medical physician linked to reason for admission

❒ Medical physician without link to reason for admission

❒ Surgeon linked to reason for admission

❒ Surgeon without link to reason for admission

❒ Other: ____________________

**External consultant:** ❒ Agrees with the decision ❒ Does not agree with the decision

**Information given to family** about WLST decision: ❒ Yes ❒ No

*If yes:* Do they support to the approach? ❒ Yes ❒ No

Do they wish to be present as their relative died? ❒ Yes ❒ No ❒ Not applicable ❒ Do not know

**Decision:**

**❒** Maximum therapeutic engagement

**❒** Withholding one or several therapies**:**

**❒** No cardiopulmonary resuscitation

**❒** No vasopressive drugs or increasement of their doses

❒ No endotracheal intubation and/or increasing inspired fraction of oxygen

❒ No renal replacement therapy

❒ No surgery or intervention (external ventricular derivation / embolization)

❒ No blood product transfusion

❒ No new antimicrobial therapy

❒ No new exams

❒ No new admission in intensive cares

**❒** Withdrawing therapies: ❒ with sedative drugs ❒ without sedative drugs

❒ Withdrawing of all the treatments

❒ Terminal extubation

❒ Setting up an inspired fraction of oxygen of 21%

❒ Withdrawing ventilation

❒ Withdrawing vasopressive drugs

❒ Withdrawing renal replacement therapy

❒ Withdrawing extra-corporeal membrane of oxygen

❒ Withdrawing hydration

❒ Withdrawing alimentation

❒ Withdrawing antibiotics

**❒** Need for a new meeting

**Patient’s outcome:** Alive at ICU discharge: ❒ Yes ❒ No

*If no:* Date of death: …../…../………

*If yes:* ❒ Patient dead between ICU discharge and D28

❒ Patient alive on D28

❒ Unknown

***Supplementary data 3.*** **WLST record note of French Anesthesiology and Intensive Care Society (SFAR^©^), translated into English by ourselves**


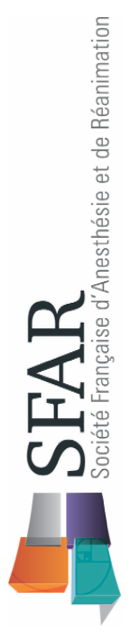


**Withholding or withdrawing life-sustaining therapeutics**

**decision sheet by la SFAR**

Patient’s identity: Date:

Physician in charge of the patient:

**Reason for initiating the discussion**

- Patient in a situation of therapeutic failure despite a well-conducted strategy and optimal care for whom withdrawing therapies aims to avoid prolonging the agony.
- Patient whose expected outcome is unfavorable in terms of prolonged life and/or quality of life, for whom withholding or withdrawing therapeutics aims to avoid unreasonable or futile treatment.
- Patient whose expected outcome is currently favorable but for whom an incrementation of treatments or a new admission in intensive cares may be unreasonnable in the event of the occurrence of a new failure.
- Other reason:

**Person participating to the discussion**

- Patient able to communicate: yes / no
- Available advance directives: yes / no / not applicable
- Medical and paramedical team:
- Trusted person:
- Others (name, grade):

**Members presents during the discussion**

- Physicians from the unit:
- Residents from the unit:
- Patient’s nurse:
- Patient’s nurse’s aid:
- Healthcare manager:
- Physicians or surgeons in charge of the patient:
- Psychologist:
- Other:

**Clinical context**

**Current organ(s) failure(s):**

- Respiratory failure
- Circulatory failure
- Neurological failure
- Kidney failure
- Liver failure
- Haematologic failure

**WLST argumentation according to the staff**

| Patient refusing a therapy of which non application could lead to death: | yes | no | NA |
| --- | --- | --- | --- |
| Existence of a possible curative strategy: | yes | no | NA |
| Clinical and paraclinic informations are sufficient: | yes | no | NA |
| Limited previous autonomy: | yes | no | NA |
| Prognosis associated to medical history: | yes | no | NA |
| Underlying illness incurable and fatal in the short-term: | yes | no | NA |
| Irreversible acute injury: | yes | no | NA |
| No improvement despite optimal active treatment: | yes | no | NA |
| Trusted person or relatives think unreasonable carrying on intensive cares: | yes | no | NA |
| Physical suffering controlled: | yes | no | NA |
| Moral suffering controlled: | yes | no | NA |
| Limited future autonomy: | yes | no | NA |
| Limited future relational quality of life: | yes | no | NA |
| Complexity of social and/or familial context: | yes | no | NA |

Comments:

**External consultant**

Name, grade:

Patient examined: Yes No

Anamnesis considered as sufficient: Yes No

WLST argumentation considered sufficient and consistent: Yes No

Decision modalities considered consistent and adapted: Yes No

Agreement with decision taken: Yes No

Comments:

**Final decision after collegial discussion**

- Therapeutic engagement level 1 : Maximum therapeutic engagement
- Engagement level 2 : Withholding one or several therapeutics
- Engagement level 3:

a. Withdrawing treatments and palliative approach without continuous deep sedation

b. Withdrawing treatments and palliative approach with continuous deep sedation

- Need of a new meeting

***Third party:*** Trusted person: Family: Relatives: Other:

- - - Are informed and consulted (identity):
    - Support the decision taken from collegial procedure.
    - Wish as their relative died: to be present ___ to be warned ___
    - Are not able to be informed.

**WLST application modalities**

| **Withholding therapies:**   - **No cardiopulmonary resuscitation** - **No treatment for a new organ failure or therapeutic incrementation** - *No vasopressive drugs* - *No endotracheal intubation* - *No NIV* - *No renal replacement therapy* - *No transfusion* - *No intracranial pressure no external ventricular derivation* - *No new antimicrobial therapy* - *No new surgery* - *No new exams* - *No more biology* | **Withdrawing therapies:**   - *Withdrawing vasopressive drugs* - *Withdrawing renal remplacement therapy* - *Instauring an inspired fraction of oxygen of 21%* - *Withdrawing mechanical ventilation* - *Withdrawing alimentation* - *Withdrawing oral fluid intake* - *Withdrawing antibiotics* - *Withdrawing of external ventricular derivation* - *Withdrawing ECMO* - *Withdrawing of all the treatments* |
| --- | --- |
| **Current withholding therapies:**   - *Inspired fraction of oxygen limited: __* - *No increaseing of mechanical ventilation (NO, prone positioning)* - *Vasopressive drugs limited at: __* | **No (new) admission in intensive cares** |

**Date of decision application:**

**Comments/evolution:**

***Supplementary data 4. FS-24R-ICU***


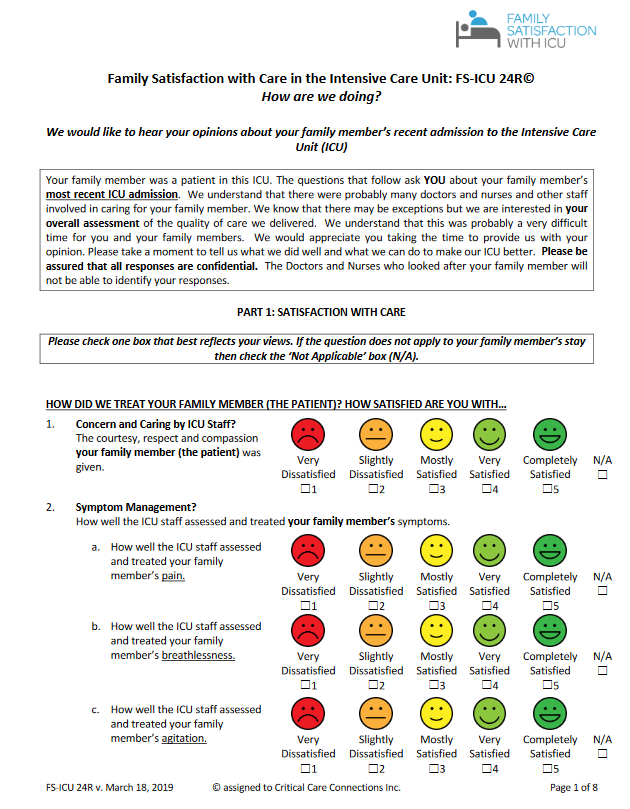


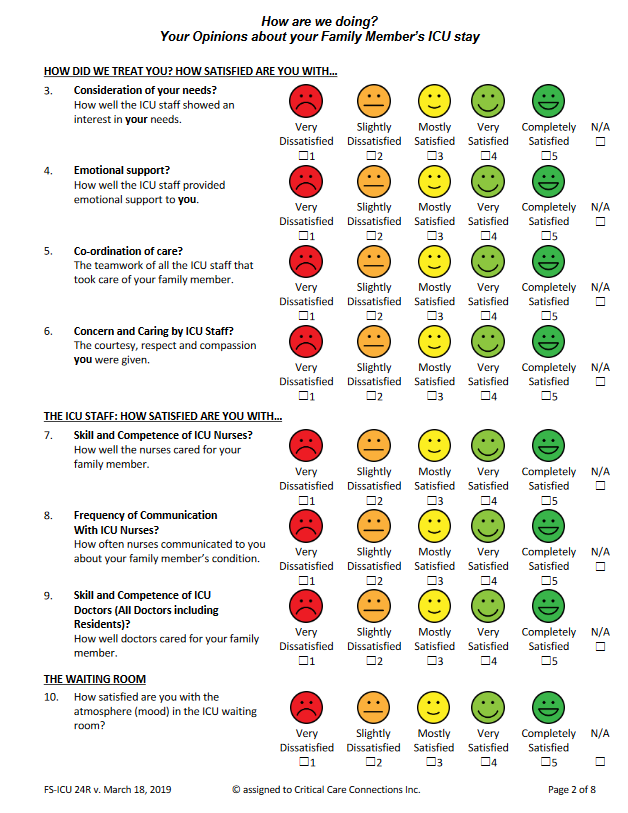


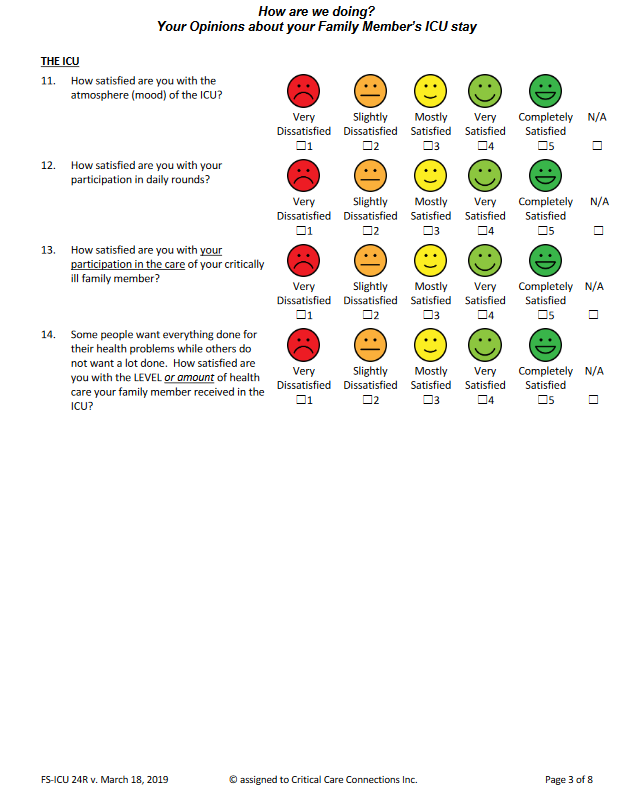


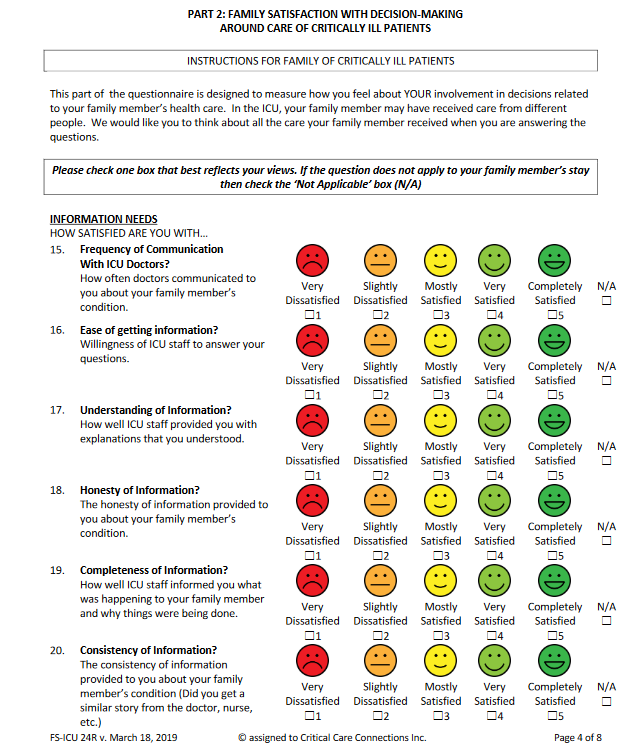


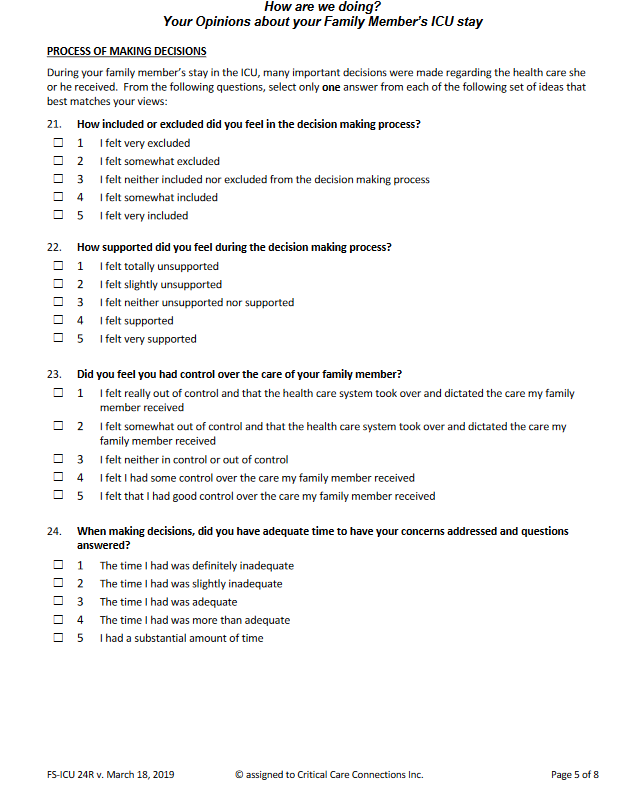


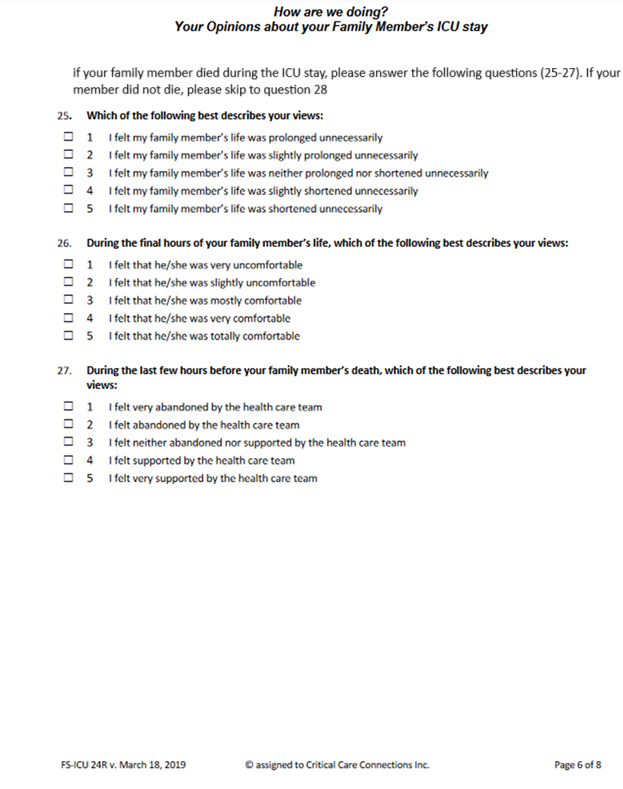


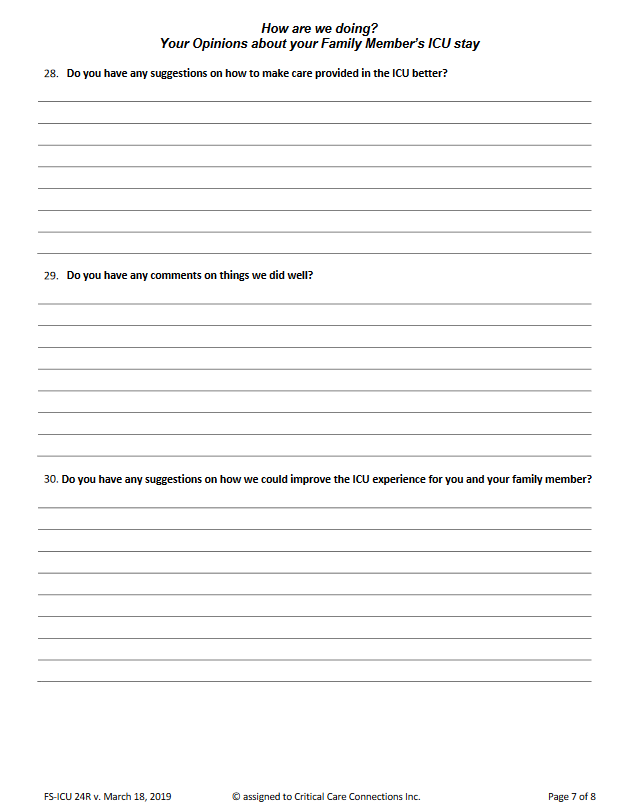


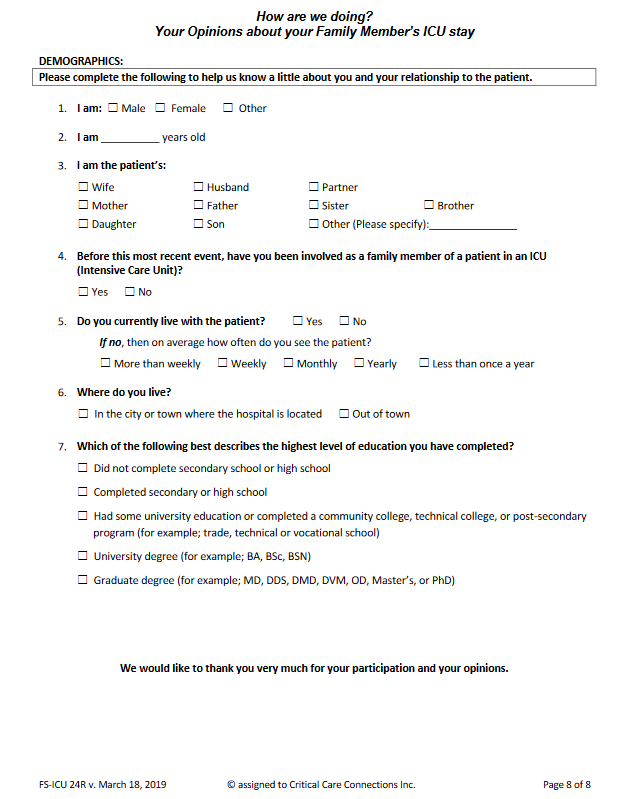


***Supplementary Data 5.* Number of inclusions and centers characteristics**

**Mean/month in the last 12 months*
